#  Escitalopram in Preschool-Age Children Diagnosed with Obsessive ‎Compulsive Disorder: A Case Report ‎

**Published:** 2016-01

**Authors:** Kemal Utku Yazici, Ipek Percinel

**Affiliations:** 1 Department of Child and Adolescent Psychiatry, Firat University Medical Faculty, ‎Elazig, Turkey. E-mail: dr.kemal.utku@outlook.com; 2 Osmaniye State Hospital, Department of Child and Adolescent Psychiatry, Osmaniye, ‎Turkey. E-mail: ipek.pr@hotmail.com

**Keywords:** Preschool Child, Obsessive-Compulsive Disorder, Escitalopram

When a literature review on pediatric obsessive-compulsive disorder (OCD) is ‎performed, it is observed that there is a dearth of research on preschool period OCD ‎cases. Although cognitive behavioral therapy is recommended as the first line of ‎treatment in preschool OCD cases when patients do not show adequate response to ‎CBT, psychopharmacological treatment offers an alternative. The first line used in ‎psychopharmacological treatment is selective serotonin reuptake inhibitors (SSRI’s). ‎However, no SSRI’s (or any other drug group) have been approved by the FDA for this ‎age group. Moreover, studies related to psychopharmacology in preschool OCD are very ‎limited in the literature, consisting mostly of case reports related to sertraline and ‎fluoxetine. In those studies, it is reported that sertraline and fluoxetine are effective in ‎preschool OCD and generally well-tolerated. In this paper, we discussed the treatment ‎and six-month follow-up period of a 3.5 year-old (42 months) female diagnosed with ‎OCD and for whom escitalopram was used. In the literature, there is a retrospective case ‎series related to this subject consisting of eleven cases, where improvement in symptoms ‎is reported with escitalopram treatment in the five of six cases diagnosed with OCD. As ‎far as we could find in literature, our paper is the second report on this subject. Our case ‎also included the youngest patient to receive escitalopram for preschool period OCD ‎and report its benefits.‎

Obsessive compulsive disorder (OCD) is a common, chronic, and treatment-resistant ‎neuropsychiatric disorder that frequently begins during childhood and adolescence (1, ‎‎^2^). Although attention has been focused on the developmental process of OCD, there ‎are few cases in the literature that discuss OCD in preschoolers (3). Cognitive-behavioral ‎therapy (CBT) is recommended as the first line of treatment in preschool OCD. ‎Psychopharmacological treatment becomes an option when CBT fails to provide ‎adequate effect. Selective serotonin reuptake inhibitor (SSRI’s) is the first choice in ‎psychopharmacological treatment (3). Research on psychopharmacological treatment in ‎preschool OCD is limited in the literature. In this paper, we discussed the six- month ‎follow-up period of a 3.5 year-old (42 months) female diagnosed with OCD, for whom ‎escitalopram was used.‎

## Case Report

‎The patient referred to our clinic because she thought she was sick when she was ‎touched and frequent hand-washing. We learned that her symptoms began three months ‎earlier and no other psychiatric application had been made. She would not let anyone, ‎including her parents, to touch her and if touched inadvertently, she reacted with crying. ‎Thinking that her toys would get dirty, she avoided playing with them or sharing them ‎with others. Evaluation of her neuro-motor development was normal, and there were no ‎features in the patient and in her family history. Physical and neurological examinations ‎were normal. Her laboratory checkup (complete blood count, liver/kidney function tests, ‎antistreptolysin-O titer, serum C-reactive protein) did not show any abnormal results. ‎The patient was diagnosed with OCD according to DSM-5 criteria. Symptoms of OCD ‎were assessed by the Childrens’ Yale-Brown Obsessive Compulsive Scale (CY-BOCS) (4). Although this scale has no validity or reliability in the Turkish population, it is ‎frequently used in Turkey (5-8). Although this scale was not designed for preschool- age ‎children, we know of no other specific method to assess preschool OCD symptoms. ‎Symptom severity and improvement in clinical follow-up were assessed by Severity (S) ‎and Improvement (I) Clinical Global Impression Scale (CGI-S, CGI-I). Functionality ‎state was assessed by Global Assessment Scale (GAS). The patient’s symptoms created ‎distress throughout the day and caused significant disturbance in functionality for both ‎herself and her family. Non-pharmacological therapy was planned originally. However, ‎because of her young age, the patient could not comply with this program, which ‎included psychoeducation and CBT. Therefore, 5 mg/day fluoxetine was initiated. On ‎the second day of the treatment, she referred to our clinic urgently because of a diffuse ‎rash on her body. After a detailed examination, it was determined that the rashes were ‎related to fluoxetine. Fluoxetine was discontinued and the rashes completely ‎disappeared within the following two days. Due to her young age, the patient was ‎unable to use drugs unless they were in a liquid form. Therefore, escitalopram was ‎initiated at 5 drops/day (2.5 mg/day). At her evaluation upon beginning the escitalopram ‎treatment, CY-BOCS score was assessed as 34, CGI-S was assessed as 5 (markedly ill) ‎and GAS was assessed as 41-50. At the control examination, two weeks following the ‎initiation of the treatment, no side effects were observed and the daily dose was ‎increased to 10 drops/day (5 mg/ day). During the examination at the eighth week of the ‎treatment, marked improvement was observed in OCD symptoms. After a five- month ‎follow-up, escitalopram was decreased to 2.5 mg/day. At the examination a month after ‎dose reduction, it was observed that her state of wellbeing was sustained (GAS was ‎assessed as 91-100). No drug-related side-effects were observed during the treatment ‎period. Case is continuing to be followed-up in our clinic. Clinical assessment scale ‎points are given in Table 1.‎

## Discussion

In this paper, a 3.5 year-old female diagnosed with OCD was treated using ‎escitalopram, and her clinical follow-up was discussed. In our study, the patient could ‎not comply with CBT and therapy could not be sustained due to her young age. While ‎treating preschool children with psychopharmacological treatment is a valid option, it ‎should only be pursued if the symptoms are severe enough to cause significant distress ‎or impair the child’s relationships or daily routine (3). Because the OCD symptoms of ‎our patient impaired functionality in both herself and her family, and caused significant ‎distress in the child's daily life, it was decided to initiate pharmacological treatment.‎

Geller et al. reported that SSRI's are more effective than placebo in the treatment of ‎pediatric OCD (9). FDA-approved SSRI's for the treatment of children and adolescents ‎OCD are sertraline (for 6 year olds and older), fluoxetine (for 7 year olds and older), ‎and fluvoxamine (for 8 year olds and older). SSRI was not approved by the FDA for ‎use in preschool cases and research on this subject in the literature was very limited. As ‎far as we could detect, there have been no randomized-controlled trials on this subject. ‎Among the limited number of case reports in the literature, fluoxetine and sertraline ‎were reported as generally well-tolerated and effective (5-8). There was only one report ‎using escitalopram. In that case series, eleven preschool patients with anxiety disorder ‎were investigated retrospectively. They did not respond to psychosocial interventions ‎and escitalopram was used in the treatment; five of the six patients who were diagnosed ‎with OCD showed anywhere from mild to very much improvements due to the ‎treatment. Ages of the patients ranged between 47-64 months old, and the dosage range ‎was 1-10 mg/day (7). ‎

It was recommended to start drug treatment in preschool cases using doses as low as ‎possible and then increase the dose as required in a controlled manner with caution, and ‎follow up carefully for side effects. Since the drugs are in a liquid form, gradually ‎increasing the dosage level from low starting doses is easily accomplished (3). In ‎Turkey, apart from fluoxetine, the only other SSRI’s molecule present in liquid ‎formulation is escitalopram. Therefore, we decided to initiate escitalopram treatment for ‎the patient. Escitalopram was started at 2.5 mg/day dosage, and was increased to 5 ‎mg/day two weeks later.‎

In a study carried out by Coskun et al., there was no response to escitalopram treatment ‎in one of the six OCD cases. In that case, drug-related behavioral disinhibition ‎developed in the second week of the treatment, so treatment was discontinued. At least ‎one side effect related to escitalopram was observed in 81.81% (n = 9) of the 11 cases ‎included in the study. Symptoms of behavioral disinhibition were reported as the most ‎frequently observed side effects (n = 5, 45.45%). No side effects were observed in ‎‎18.8% (n = 2) of the cases (7). Symptoms of behavioral disinhibition have also been ‎frequently observed in other case reports in the literature with respect to the ‎psychopharmacological treatment of preschool OCD (5, 6 and 8). In another study, SSRI ‎tolerances of 39 patients under seven years of age were investigated; and it was reported ‎that six of the patients could not continue on SSRI treatment due to behavioral ‎activation (10). In contrast to the results of those studies, there were no side effects in ‎our case that could be related to escitalopram use.‎

It is reported in the literature that early-onset OCD is associated with worse prognosis ‎‎ (11). Therefore, effectively treating the disorder at an early stage is critical. To the best ‎of our knowledge, our case is the second report in the literature on this subject. ‎Additionally, it is observed that our case is the youngest preschool OCD case who was ‎treated with escitalopram and had benefited it from treatment. Our findings are ‎important with regards to drawing attention to lack of research on this subject. The ‎results of this case report indicate that there should be further randomized, controlled ‎studies with larger sample sizes on the treatment of early-onset OCD patients.‎

**Table 1:** Clinical Rating Scale Scores at 4 Week Intervals

	**CY-BOCS**	**CGI-S**	**CGI-I**	**Stage of Response** *
I. Rating (Baseline)	34	5	-	-
II. Rating	29	5	4	V
III. Rating	24	4	3	IV
IV. Rating	17	2	2	III
V. Rating	13	2	2	II
VI. Rating	8	2	1	II
VII. Rating (Endpoint)	3	1	1	I

CY-BOCS: Childrens’ Yale-Brown Obsessive Compulsive Scale CGI-S: Clinical Global Impression Scale-Severity CGI-I: Clinical Global Impression Scale-Improvement

* Pallanti and Quercioli (2006) (12); V: Non-Response, decrease in YBOCS scores is less than 25% and CGI-4 IV:Partial Response, decrease in YBOCS scores is greater than 25% and less than 35% III: Full Response, decrease in YBOCS scores is greater than 35% and CGI-S 1 or 2 II: Remission, YBOCS score <16 I: Recovery, YBOCS score <8
